# Composite Filament Materials for 3D-Printed Drone Parts: Advancements in Mechanical Strength, Weight Optimization and Embedded Electronics

**DOI:** 10.3390/ma18112465

**Published:** 2025-05-24

**Authors:** Antreas Kantaros, Christos Drosos, Michail Papoutsidakis, Evangelos Pallis, Theodore Ganetsos

**Affiliations:** Department of Industrial Design and Production Engineering, University of West Attica, 12244 Athens, Greece

**Keywords:** composite filaments, 3D printing, UAV fabrication, carbon-fiber-infused materials, high-speed FFF, additive manufacturing, drone optimization, structural integrity, embedded electronics, hybrid manufacturing

## Abstract

The rapid advancement of 3D printing technologies has greatly assisted drone manufacturing, particularly through the use of composite filaments. This paper explores the impact of fiber-reinforced materials, such as carbon-fiber-infused PLA, PETG, and nylon, on the mechanical performance, weight optimization, and functionality of unmanned aerial vehicles (UAVs). The study highlights how additive manufacturing enables the fabrication of lightweight yet structurally robust components, enhancing flight endurance, stability, and payload capacity. Key advancements in high-speed fused filament fabrication (FFF) printing, soluble support materials, and embedded electronics integration are examined, demonstrating their role in producing highly functional UAV parts. Furthermore, the challenges associated with material processing, cost, and scalability are discussed, along with solutions such as advanced extruder designs and hybrid manufacturing approaches that combine 3D printing with CNC machining. By utilizing composite filaments and innovative fabrication techniques, 3D printing continues to redefine drone production, enabling rapid prototyping and on-demand customization. The use of carbon-fiber-infused PLA, PETG, and nylon has demonstrated outstanding improvements in strength-to-weight performance, structural durability, and dimensional stability—key factors for enhancing flight endurance, maneuverability, and payload capacity in UAV applications. These composite materials also support the integration of embedded electronics and functional features, reinforcing their suitability for high-performance drone parts. Looking forward, future research should explore the potential of nanocomposite filaments not as a replacement but as a complementary advancement to existing composites. These materials offer opportunities for further enhancing multifunctionality, such as thermal/electrical conductivity and in situ sensing, which could expand UAV capabilities significantly.

## 1. Introduction

The performance and efficiency of unmanned aerial vehicles (UAVs) are heavily dependent on the materials used in their construction. Drones require a delicate balance between optimal mechanical behavior and weight, as excessive weight reduces flight time and agility, while insufficient mechanical behavior compromises structural integrity and durability [1]. The optimization of materials is particularly critical for applications where endurance, speed, and payload capacity are key performance metrics, such as in surveillance, delivery services, and search-and-rescue operations [2,3].

Lightweight materials, such as polymer-based composites, play a crucial role in enhancing UAV efficiency by minimizing energy consumption and maximizing lift-to-weight ratios [4]. Traditional drone manufacturing has relied on materials like aluminum and fiberglass composites, but the introduction of advanced 3D printing technologies has enabled the use of high-performance thermoplastics reinforced with fibers such as carbon, glass, or Kevlar [5]. These composite materials offer superior mechanical properties, including high tensile strength, stiffness, and resistance to environmental degradation, making them ideal candidates for UAV applications [6].

Furthermore, the integration of lightweight, strong materials is vital in reducing vibrations and improving aerodynamics, leading to enhanced flight stability and maneuverability [7]. In high-speed or high-load scenarios, such as racing drones or industrial UAVs carrying specialized sensors, material selection becomes even more crucial. The ability to fabricate drone components with customizable mechanical properties through 3D printing allows for structural optimization, reducing weight while maintaining robustness [8].

With the rapid advancement of composite filaments in additive manufacturing, drone fabrication is shifting towards more efficient and sustainable production methods. The development of carbon-fiber-infused thermoplastics, in particular, has opened new possibilities for manufacturing UAV components that rival traditionally machined counterparts in terms of both performance and longevity [9]. As the demand for lightweight yet durable drone components continue to grow, leveraging the advantages of modern composite materials will be key to advancing UAV design and functionality.

While this work focuses primarily on composite filament materials used in additive manufacturing, it is important to contextualize these advancements against traditional materials commonly employed in drone manufacturing—namely, metals and metal alloys such as aluminum, titanium, and magnesium. These materials are favored for their high strength, thermal stability, and fatigue resistance, making them suitable for high-performance UAV components such as frames, motor housings, and landing gear [10]. However, despite their excellent mechanical properties, metallic components often come at the cost of increased weight and reduced design flexibility. In contrast, polymer-based composites, especially those reinforced with carbon, glass, or aramid fibers, offer comparable strength-to-weight ratios while enabling lightweight construction, reduced energy consumption, and improved flight performance [11]. Moreover, composite filaments allow for the integration of complex geometries and embedded functionalities, which are difficult to achieve with traditional subtractive or forming processes used for metals. Thus, choosing composite materials in drone manufacturing is dictated not only by material performance but also by the broader benefits offered by additive manufacturing in terms of customization, rapid prototyping, and structural optimization.

Beyond extrusion-based 3D printing, conventional manufacturing techniques such as injection molding, vacuum forming, and CNC machining have long played a pivotal role in the mass production of drone components. Injection molding, for example, is widely used to fabricate plastic airframes and structural elements with high repeatability and dimensional precision, especially for consumer-grade drones [12]. Vacuum forming is also employed for lightweight covers, enclosures, and aerodynamic shells due to its low tooling cost and suitability for thin-walled parts [13]. CNC machining, particularly with aluminum alloys, remains the standard for high-performance or custom-built drones requiring tight tolerances and superior mechanical properties. While these traditional techniques remain integral to the UAV industry, they often involve longer lead times, higher upfront tooling costs, and limited design flexibility compared to additive manufacturing [14]. The emergence of high-speed fused filament fabrication (FFF) and composite filament materials now offers a competitive alternative, particularly for applications requiring rapid iteration, weight optimization, and integration of embedded systems. As such, additive manufacturing complements and, in some contexts, competes with established fabrication techniques in the evolving landscape of drone production.

**Figure 1:**
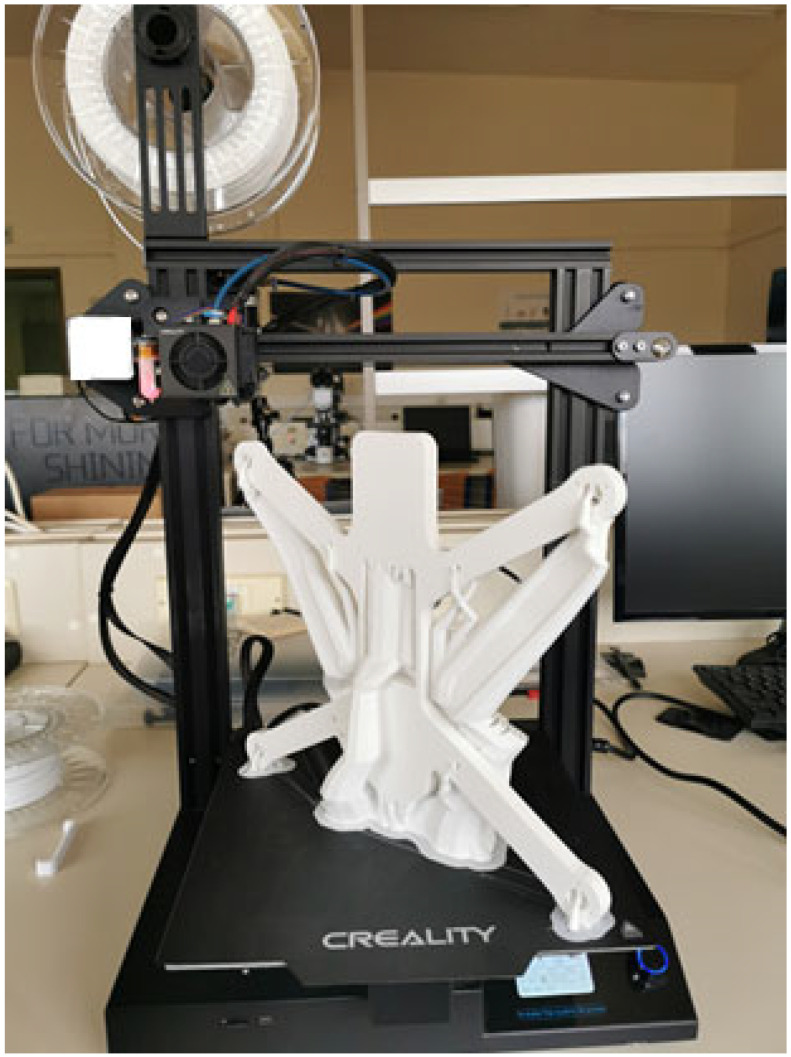
Drone frame upon its fabrication process completion in a desktop FFF 3D printer (Shenzhen Creality 3D technology Co., Ltd., Shenzhen, China).

**Figure 2:**
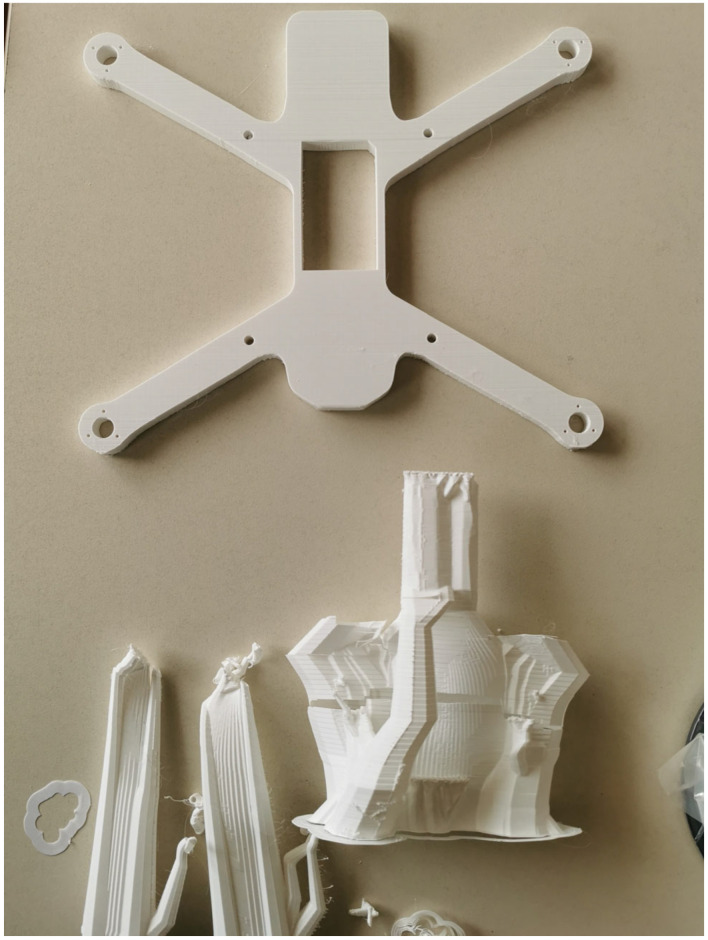
Drone frame upon the support structures’ removal process completion.

Additive manufacturing has emerged as a novel enabling technology in the aero-space and unmanned aerial vehicle (UAV) industries, offering unprecedented design flexibility, material efficiency, and production scalability [15]. Unlike traditional subtractive manufacturing techniques, which often involve significant material waste and long lead times, 3D printing enables the rapid fabrication of complex, lightweight drone components with minimal waste. This is particularly advantageous in UAV design, where weight reduction directly translates into improved flight endurance, agility, and energy efficiency. Through layer-by-layer deposition, 3D printing allows for the creation of intricate geometries, such as aerodynamic fuselages, lattice-structured frames, and integrated wiring channels, which would be challenging or impossible to achieve using conventional manufacturing methods [16]. Additionally, the ability to rapidly prototype and iterate designs accelerates the development cycle of drone technologies, facilitating real-time testing and optimization for enhanced performance and structural integrity [17,18]. Figure 1 depicts a drone frame upon its fabrication process completion in a desktop FFF 3D printer, while Figure 2 depicts the drone frame upon the support structures’ removal process completion.

Apart from prototyping, 3D printing has become increasingly viable for end-use drone production due to advancements in material science and high-speed additive manufacturing techniques [19,20]. Moreover, high-speed fused filament fabrication (FFF) systems, equipped with improved extruder designs and heated build chambers, enable the production of large, high-strength parts with superior layer adhesion and reduced anisotropy [21]. Another key advantage of additive manufacturing in UAV production is its ability to facilitate distributed manufacturing, allowing drone components to be fabricated on-demand and in close proximity to deployment sites [22,23]. This is particularly relevant for disaster response, military operations, and remote research applications, where logistical constraints make traditional supply chain models inefficient.

The integration of carbon fiber into thermoplastic filaments has revolutionized the landscape of additive manufacturing, particularly in applications demanding high strength-to-weight ratios, such as drone fabrication. Carbon-fiber-infused filaments combine the lightweight nature of polymer matrices with the exceptional mechanical properties of carbon fibers, resulting in materials that exhibit enhanced tensile strength, stiffness, and resistance to environmental stressors [24]. Unlike standard 3D printing filaments, which often suffer from limited structural integrity under mechanical loads, carbon-fiber-reinforced composites provide significantly improved rigidity and durability, making them ideal for fabricating UAV components such as airframes, motor mounts, and propellers [25]. The inclusion of short or continuous carbon fibers within filaments such as PLA, PETG, or nylon not only increases mechanical performance but also reduces thermal expansion and warping during the printing process, leading to greater dimensional stability and precision [26]. These characteristics are particularly crucial in UAV applications, where even minor deviations in structural geometry can impact aerodynamics, flight stability, and overall performance.

Beyond their mechanical advantages, carbon-fiber-infused filaments hold significant potential in advancing UAV manufacturing through their compatibility with high-speed additive manufacturing techniques [27]. With the development of next-generation fused filament fabrication (FFF) systems capable of processing reinforced thermoplastics at elevated speeds, drone production can now achieve both efficiency and structural optimization without compromising quality [28]. Moreover, the anisotropic nature of carbon fiber reinforcement enables designers to intentionally orient fiber alignment within printed parts, further optimizing load-bearing capabilities for specific drone applications [29]. In addition, ongoing research into hybrid composite filaments—such as those incorporating graphene or carbon nanotubes—aims to further enhance electrical conductivity and multifunctionality, leading to UAV components with embedded sensing and structural health monitoring capabilities [30]. In this way, as 3D printing technologies continue to advance, the introduction of carbon-fiber-infused filaments represents a critical step toward the development of lightweight, high-performance UAVs with improved durability, energy efficiency, and adaptability across diverse operational environments [31,32].

Advancements in high-speed fused filament fabrication (FFF) printing have greatly aided the production of drone components by enabling faster fabrication processes without sacrificing the precision or quality of the parts. High-speed FFF printers, such as those developed by Bambu Labs (Shenzhen, China), employ advanced motion systems and optimized extrusion technologies to increase printing speeds while maintaining high resolution and accuracy [33]. This enhancement allows for rapid prototyping and production of drone parts, which is particularly advantageous in industries that require frequent design iterations or low-volume manufacturing. The speed at which high-speed FFF printers can fabricate parts has the potential to reduce the time from design to production significantly, improving the efficiency of the development cycle for drone manufacturers. Additionally, the ability to print with high-performance materials such as carbon-fiber-infused filaments further enhances the strength-to-weight ratio of the components, making them suitable for the demanding operational conditions drones often face.

In addition to speed, the integration of soluble support materials has greatly expanded the design possibilities for 3D-printed drone parts. Soluble support materials, such as polyvinyl alcohol (PVA) or polyethylene glycol (PEG), are used to create temporary scaffolds that can be easily dissolved in water or a chemical solution after printing, leaving behind complex, intricate structures that were previously difficult to achieve with traditional support methods [34,35]. This technology allows for the creation of geometrically complex components, including internal cavities, integrated sensor mounts, and other functional features that enhance the drone’s performance [36]. The use of soluble supports is particularly beneficial for parts with internal electronics or sensor integration, as it ensures that the supports do not interfere with the functionality or wiring of the components [37]. The combination of high-speed FFF printing and soluble support materials enables the fabrication of lightweight, highly functional drone parts, contributing to the overall advancement of drone manufacturing and broadening the scope of what can be achieved with 3D printing technologies.

In conclusion, the increasing demand for lightweight, durable, and functional drone components has driven significant advancements in 3D printing technologies, particularly through the development of composite filaments. This chapter has highlighted the importance of material optimization in drone fabrication, emphasizing the role of carbon-fiber-infused filaments in enhancing strength-to-weight ratios. It also described key innovations such as high-speed FFF printing and soluble support materials, which have expanded the design possibilities for complex, functional drone parts. In the continuation of this work, the subsequent chapters will look into the specific types of composite filaments used in drone manufacturing, examining their mechanical properties, applications, and optimization techniques. The discussion will also cover the integration of embedded electronics, the role of high-speed printing technologies, and the challenges faced in material processing and scalability. Ultimately, this work aims to provide a comprehensive overview of how advancements in 3D printing are transforming the fabrication of drones, enabling more efficient production, improved performance, and the incorporation of innovative designs and functionalities.

## 2. Overview of Composite Filaments in 3D Printing

### 2.1. Definition and Classification of Composite Filaments

Composite filaments are a specialized class of 3D printing materials that incorporate a base thermoplastic polymer (matrix), such as PLA, ABS, or PETG, combined with reinforcing materials like carbon fiber, glass fiber, or Kevlar [12]. These fibers are typically added in small, controlled amounts to enhance the mechanical properties of the filament, enabling the production of parts with improved strength, stiffness, and impact resistance compared to unreinforced thermoplastics [38]. The base polymer (matrix) provides the necessary structural foundation, while the reinforcement material contributes specific desired attributes that make the filament suitable for demanding applications, such as drone fabrication. The integration of these fibers also affects the material’s thermal stability, wear resistance, and dimensional stability, addressing common challenges in industries that require high-performance components [39]. Composite filaments are essential for applications where traditional 3D printing materials would not meet the necessary mechanical or functional demands, particularly in industries like aerospace, automotive, and robotics.

The classification of composite filaments is primarily based on the type of reinforcing material used. As priorly mentioned, carbon-fiber-infused filaments are among the most widely used due to their exceptional strength-to-weight ratio, making them ideal for applications that require lightweight yet strong parts [40]. These filaments are particularly suitable for drone components, as they provide the necessary structural integrity without adding significant weight, thus enhancing overall flight performance. Glass-fiber-infused filaments, on the other hand, offer higher impact resistance and stiffness, making them appropriate for parts that must endure mechanical stress and environmental wear [41,42]. Kevlar-infused filaments are known for their superior abrasion resistance and flexibility, which are useful in parts exposed to high wear or stress, such as protective enclosures [43,44,45]. Additionally, some composite filaments combine multiple reinforcement types to achieve a balanced set of properties, tailoring the material to the specific requirements of the application [45,46]. The selection of the right composite filament depends on the performance characteristics needed for the final product, as well as factors like printability, cost, and environmental considerations. Understanding the classification and properties of these composite filaments is essential for optimizing their use in 3D printing applications, particularly for the development of advanced drone components. Table 1 presents a classification of composite filaments used in 3D printing, including key properties and general applications across industries.

### 2.2. Comparison of Carbon-Fiber-, Glass-Fiber-, and Kevlar-Infused Filaments for Drone Parts

Carbon fiber, glass fiber, and Kevlar-infused filaments are all widely used in the 3D printing of drone components, each offering distinct mechanical advantages depending on the specific requirements of the application [47]. Carbon-fiber-infused filaments are known for their exceptional strength-to-weight ratio, making them ideal for drone parts where weight reduction is critical for maximizing flight performance [48]. The incorporation of carbon fiber into a polymer matrix increases the material’s stiffness and structural integrity without significantly adding to its weight, thus contributing to the overall efficiency of the drone [49]. Carbon fiber filaments are particularly advantageous for structural components, such as the drone frame, arms, and motor mounts, where strength and rigidity are paramount [50]. Furthermore, these filaments often exhibit improved thermal stability, which is beneficial in high-temperature environments [51]. However, carbon fiber filaments tend to be more brittle compared to other fiber-reinforced materials, which can be a drawback for parts subjected to high impact or sudden stress [52,53].

**Figure 3:**
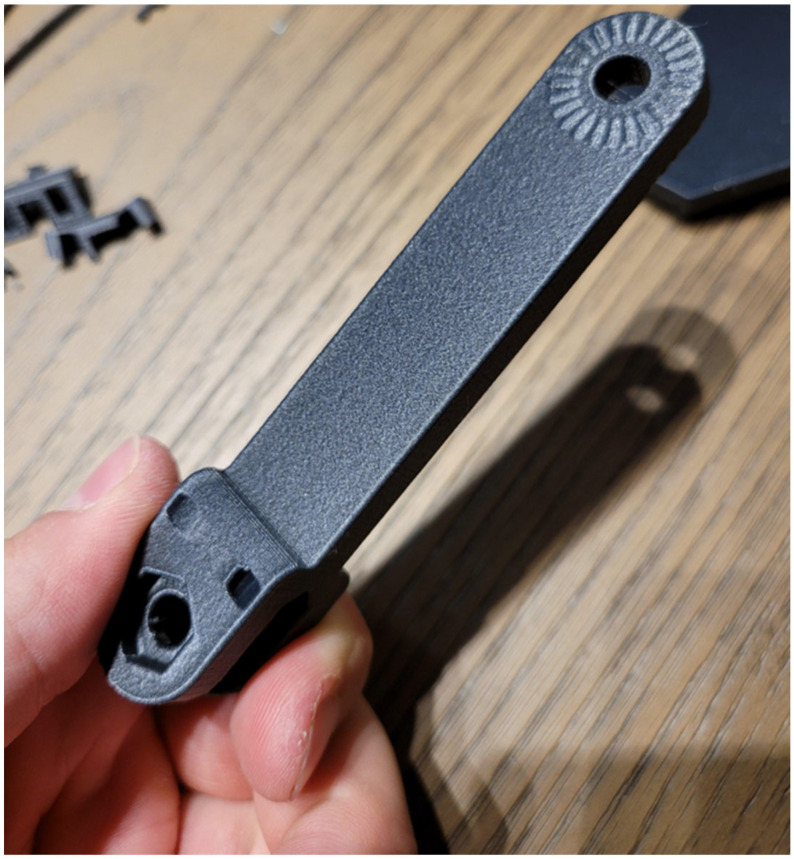
Three-dimensional printed part made out of carbon-fiber-reinforced PLA material.

Glass-fiber-infused filaments, on the other hand, provide superior impact resistance and increased stiffness, making them suitable for parts that must withstand mechanical stress or rough handling [54]. The fibers, often embedded in a base polymer such as PLA or ABS, significantly enhance the material’s ability to absorb impact energy, making them well-suited for drone components like propeller housings, landing gear, and protective panels [55]. Glass fiber filaments also offer better durability and resistance to wear and tear compared to carbon fiber, which is crucial for components exposed to harsh environmental conditions [56]. However, while glass fiber provides excellent mechanical strength, it is heavier than carbon fiber, which can impact the overall weight of the drone. Kevlar-infused filaments, known for their exceptional abrasion resistance and toughness, offer a unique combination of flexibility and strength [57]. Kevlar is especially effective in parts that need to endure extreme wear, such as protective casings, cables, or landing gear [58]. Although it provides greater durability in terms of resistance to abrasion and impact, Kevlar-infused filaments are generally more difficult to print with and require specialized equipment due to their high friction and potential for clogging the printer nozzle [59]. Overall, the choice between these composite filaments depends on the specific demands of the drone part being produced, with carbon fiber excelling in strength-to-weight optimization, glass fiber offering durability and impact resistance, and Kevlar providing superior abrasion and wear resistance. Figure 3 shows a 3D printed part made out of carbon-fiber-reinforced PLA material.

### 2.3. Influence of Fiber Content, Dispersion, and Bonding on Mechanical Properties

The fiber content in composite filaments plays a significant role in determining the mechanical properties of the final printed part [60]. As the fiber content increases, the material typically exhibits enhanced strength, stiffness, and thermal stability [60]. However, the relationship between fiber content and mechanical performance is not linear, as excessively high fiber concentrations can lead to issues such as reduced printability, material brittleness, and a higher likelihood of defects during the printing process [61]. Optimal fiber content is critical for achieving a balance between enhanced mechanical performance and processability [62]. For example, in carbon-fiber-reinforced filaments, a fiber content ranging from 15% to 30% by weight is commonly used to achieve superior strength and stiffness without compromising the filament’s ability to flow smoothly through the extruder and adhere to the print bed [63]. At higher fiber percentages, the material may become more prone to nozzle clogging or excessive wear on the printer’s components [64,65]. Therefore, careful control of fiber content is necessary to ensure that the printed part exhibits the desired mechanical properties without introducing new challenges during the fabrication process.

The dispersion of fibers within the polymer matrix is another critical factor influencing the mechanical properties of composite filaments [66]. Uniform dispersion of the reinforcing fibers is essential for achieving consistent performance throughout the printed part [67]. Poor dispersion, where fibers tend to agglomerate or form clumps, can result in localized weak points that compromise the structural integrity of the part. This is particularly important in materials like carbon fiber and glass fiber filaments, where uneven fiber distribution can lead to anisotropic behavior—where the material’s properties differ depending on the direction of load application [68]. The proper dispersion of fibers ensures that the reinforcement is effective in all areas of the part, allowing the material to exhibit consistent strength, stiffness, and impact resistance [69]. Advanced manufacturing techniques, such as high-shear mixing or melt extrusion, are often employed during the filament production process to achieve optimal fiber dispersion, which is crucial for maintaining the desired mechanical properties of the final printed component [70,71].

Bonding between the reinforcing fibers and the base polymer is another key factor influencing the mechanical performance of composite filaments [72]. The effectiveness of this bonding directly impacts the transmission of stress between the fiber and the matrix, as well as the overall strength and durability of the printed part. Inadequate bonding can lead to poor load transfer, resulting in fiber pull-out or delamination under stress [73]. This issue is particularly prevalent in materials with insufficient surface treatment on the fibers or incompatible polymer matrices [74]. To improve bonding, surface modifications such as chemical treatments, plasma coating, or sizing agents are often applied to the fibers before they are incorporated into the filament. These modifications enhance the interaction between the polymer matrix and the fibers, improving mechanical properties such as tensile strength, shear strength, and impact resistance [75]. Additionally, the quality of the bonding can be influenced by the printing process itself, including factors such as extrusion temperature, print speed, and layer adhesion. Proper control of these parameters ensures that the fibers are effectively bonded to the polymer matrix, allowing the composite material to perform as intended under real-world conditions.

Beyond fiber content, dispersion, and bonding, the anisotropic behavior of 3D-printed composite materials is a critical factor influencing mechanical performance. Due to the layer-by-layer nature of the printing process, these materials exhibit directional mechanical properties, often resulting in weaker interlayer adhesion compared to in-plane strength. This directional dependency can lead to stress concentrations and increased risk of delamination under mechanical loads applied perpendicular to the print layers. Factors such as the volume fraction and aspect ratio of the reinforcing fibers significantly affect this behavior [76]. Higher fiber content can enhance strength and stiffness in the print plane but may contribute to brittleness or poor interlayer cohesion if not properly optimized. Similarly, longer fibers tend to improve load transfer and mechanical reinforcement, but can pose challenges in achieving uniform dispersion, potentially creating weak spots [77]. Additionally, the formation of voids within the printed structure—resulting from incomplete fusion, fiber misalignment, or processing inconsistencies—can act as initiation points for mechanical failure. The size, distribution, and location of these voids play a critical role in determining the overall structural integrity and durability of the printed part [78]. Addressing these interrelated factors is essential for improving the reliability, performance, and application scope of fiber-reinforced composite filaments in high-demand environments such as drone manufacturing.

## 3. Strength-to-Weight Optimization in Drone Components

### 3.1. Analysis of Mechanical Properties: Tensile Strength, Stiffness, and Impact Resistance

The mechanical properties of 3D-printed drone components are critical for ensuring optimal performance, durability, and safety [79]. These properties, particularly tensile strength, stiffness, and impact resistance, directly influence the structural integrity of the drone frame and its ability to withstand external stresses encountered during flight [80]. As drones are subject to dynamic loading, vibration, and potential impact forces, understanding and optimizing these mechanical properties is paramount to their operational efficiency [81,82].

Tensile strength refers to the maximum stress that a material can withstand while being stretched or pulled before breaking. In drone fabrication, composite filaments, particularly carbon-fiber-infused materials, are highly regarded for their superior tensile strength compared to conventional thermoplastics [83]. The addition of carbon fibers enhances the matrix’s ability to resist elongation, resulting in lightweight yet robust components capable of handling high tension loads. This property is crucial for drone arms, landing gear, and other structural elements subjected to tensile forces during flight maneuvers and in emergency landings [84].

Stiffness, often quantified as the material’s Young’s modulus, is a measure of its resistance to deformation under applied stress [85]. Stiffness is particularly important for maintaining the shape and stability of drone frames under operational loads. Composite filaments like carbon fiber PLA and PETG exhibit significantly improved stiffness compared to non-reinforced polymers, making them ideal candidates for structural parts where minimal flexing is desired [86]. A high stiffness-to-weight ratio ensures that the drone remains stable during flight, especially at high speeds or when carrying heavy payloads, without excessive bending or deformation [87].

Impact resistance is the material’s ability to absorb and dissipate energy when subjected to sudden forces or impacts [88]. For drones, impact resistance is crucial in preventing damage during crashes or collisions with obstacles [89]. Carbon fiber composites, while offering high strength and stiffness, also exhibit enhanced impact resistance due to the reinforcement fibers, which help to distribute and absorb the energy of impacts [90]. However, the level of impact resistance can be influenced by the filament’s orientation, fiber distribution, and the inherent brittleness of certain matrix materials. For instance, while carbon fiber PLA offers excellent strength and stiffness, its brittleness can be a limiting factor in terms of impact resistance, especially under low-temperature conditions [91].

### 3.2. Case Studies of Carbon Fiber PLA, PETG, and Nylon in Drone Applications

The application of carbon-fiber-reinforced filaments such as PLA, PETG, and nylon in drone manufacturing has demonstrated significant improvements in structural integrity, weight reduction, and mechanical performance. The published study by Prado [92] highlights the role of topology optimization in redesigning UAV airframes for industrial applications. By utilizing fused deposition modeling (FDM) and composite filaments, this study successfully developed a lightweight yet robust airframe that integrates electronic components and landing systems efficiently. The incorporation of carbon fiber composites enhanced the mechanical properties of the UAV while ensuring cost-effectiveness, making additive manufacturing a viable alternative to conventional molding techniques [92]. The use of PETG-carbon fiber blends provided durability and impact resistance, while PLA-based composites maintained ease of fabrication and economic feasibility.

Further supporting these findings, the published literature work presented by Mihailescu et al. [93] explores the practical implementation of carbon-fiber-reinforced epoxy composites in drone airframe construction. Unlike PLA and PETG composites, this study focused on the integration of epoxy-based carbon fiber materials alongside additive manufacturing techniques, demonstrating the feasibility of hybrid composite fabrication. The study involved iterative design refinements using Inventor 2023 software (Autodesk, San Francisco, CA, USA), resulting in an optimized prototype with enhanced mechanical properties. Notably, tensile and bending tests confirmed the material’s superior strength-to-weight ratio, making it an ideal candidate for UAV structural components such as main frames, motor mounts, and landing gear [93]. These results reinforce the advantages of using carbon-fiber-based filaments, particularly when strength, stiffness, and weight minimization are critical design constraints in UAV development.

Moreover, the published literature work presented by Šostakaite et al. [94] examined the suitability of PLA and lightweight PLA in UAV production, particularly in the fabrication of a flying wing-type drone. The investigation found that while PLA-based drones are cost-effective and easily manufacturable using desktop 3D printers, their mechanical limitations necessitate further optimization. The study explored lightweight PLA, which exhibits density variations based on printing temperature, leading to improved aerodynamic performance without sacrificing mechanical integrity [94]. Simplified bending tests demonstrated significant reserves in mechanical resistance, confirming that PLA can serve as a viable material for UAV prototyping. However, durability concerns and fragmentation risks associated with printing limitations highlight the necessity of reinforcing PLA with carbon fibers or transitioning to more advanced materials such as PETG or nylon composites for mission-critical UAV applications.

Advancements in carbon-fiber-reinforced materials for drone manufacturing were further validated by the published literature work presented by Šančić et al., [3]. This study compared different additive manufacturing techniques, including FDM, selective laser sintering (SLS), and continuous fiber fabrication (CFF), revealing that CFF-produced parts exhibited mechanical properties at least four times higher than those manufactured via FDM or SLS [3]. The results indicated that by increasing the fiber content within the polymer matrix, the structural integrity of UAV airframes could rival that of aluminum alloys. Stress–strain analysis confirmed that carbon-fiber-infused nylon and PETG significantly outperformed standard polymeric filaments in tensile strength and impact resistance, supporting their use in high-performance drone applications.

Finally, the published literature work presented by Palmer and Laliberte [20], investigated the structural benefits of carbon-fiber-reinforced filaments in drone propulsion systems. The study found that carbon-fiber-reinforced PETG and nylon propellers exhibited a 20% increase in impact resistance compared to their non-reinforced counterparts, while non-planar slicing techniques improved performance by an additional 65% [20]. These findings suggest that by optimizing print orientation and material composition, the mechanical limitations of traditional 3D-printed propellers can be mitigated, making them viable alternatives to conventionally manufactured drone components.

Collectively, these case studies emphasize the transformative potential of carbon-fiber-reinforced PLA, PETG, and nylon in UAV manufacturing. By grasping the mechanical benefits of composite filaments, 3D-printed drones can achieve superior structural efficiency, weight optimization, and adaptability, making them highly suitable for diverse applications ranging from industrial inspections to autonomous aerial operations.

### 3.3. Finite Element Analysis (FEA) and Experimental Testing for Optimizing Structures

Finite Element Analysis (FEA) and experimental testing play a crucial role in optimizing the structural performance of 3D-printed drone components, particularly those fabricated using carbon-fiber-reinforced filaments such as PLA, PETG, and nylon [95]. FEA enables engineers to simulate mechanical stresses, deformations, and load distributions across UAV airframes and propellers, facilitating data-driven design improvements before physical manufacturing. By applying numerical methods to analyze material behavior under varying operational conditions, FEA helps predict structural failure points, optimize weight distribution, and enhance mechanical properties [96]. The integration of anisotropic material modeling within FEA is particularly relevant for composite filaments, as carbon fiber reinforcements exhibit directional strength variations. For example, in UAV arm structures, FEA simulations can identify the most efficient fiber orientation and infill patterns to maximize tensile and impact resistance while minimizing weight. These predictive capabilities reduce the need for iterative prototyping, saving both time and material costs in drone development [97]. Furthermore, FEA can assess thermal expansion and layer adhesion in fused filament fabrication (FFF) prints, addressing warping issues and ensuring dimensional stability for mission-critical UAV components.

Despite the predictive ability of FEA, experimental testing remains essential for validating the structural integrity of 3D-printed drone components, particularly in dynamic flight environments [97]. Experimental methodologies such as tensile, compression, and bending tests allow researchers to compare simulation data with real-world mechanical performance, ensuring the accuracy of FEA models. In studies evaluating carbon-fiber-infused PETG and nylon, tensile strength tests have demonstrated significant improvements over standard polymeric materials, reinforcing FEA predictions [98]. Additionally, impact resistance tests on drone airframes and propellers printed with carbon fiber additives have revealed increased durability under sudden loads, such as crash landings or high-speed impacts. One particularly insightful study focused on multirotor UAV airframes produced via continuous fiber fabrication (CFF), where experimental stress–strain analysis showed that fiber-reinforced specimens exhibited up to four times higher maximum stresses compared to FDM and SLS-manufactured parts. These findings align with FEA simulations, confirming that higher fiber content contributes to enhanced load-bearing capacity. By combining FEA and physical testing, engineers can fine-tune UAV designs to withstand real-world operational stresses while maintaining an optimal strength-to-weight ratio [99].

The synergy between FEA and experimental validation extends beyond structural testing to include aerodynamic performance and fatigue analysis, which are critical for long-term UAV reliability. FEA-based computational fluid dynamics (CFD) simulations can predict airflow characteristics around drone frames, optimizing aerodynamics for improved flight efficiency [100]. Experimental wind tunnel testing then corroborates these results, ensuring minimal turbulence and drag [101,102]. Similarly, fatigue testing assesses how cyclic loading affects 3D-printed UAV structures over time, revealing potential failure points that may not be immediately evident in static FEA simulations [103,104]. By incorporating both computational and experimental approaches, UAV manufacturers can iteratively refine their designs, achieving superior mechanical resilience and operational efficiency. The ongoing advancements in high-speed FEA solvers and real-time experimental monitoring further streamline this process, enabling rapid optimization of drone structures for diverse applications such as industrial inspections [105], aerial mapping [106], and autonomous surveillance [107,108]. As additive manufacturing technologies evolve, the integration of FEA and experimental testing will remain indispensable in ensuring the performance, safety, and reliability of 3D-printed UAVs. Table 2 depicts the mechanical properties of composite filaments for 3D-printed drone parts.

## 4. High-Speed FFF 3D Printing for Drone Fabrication

### 4.1. Evolution of High-Speed 3D Printing Technologies

The rapid evolution of high-speed fused filament fabrication (FFF) 3D printing has significantly influenced the manufacturing of drone components by enhancing production efficiency, precision, and material performance [36]. Traditional FFF systems have long been limited by print speeds of around 50–100 mm/s, constrained by material flow dynamics, thermal stability, and mechanical layer adhesion. However, the emergence of high-speed 3D printers, such as Bambu Labs’ X1 (Bambu Labs, Shenzhen, China) series, has redefined the achievable throughput by integrating advanced motion systems, reinforced extruders, and optimized thermal management. These advancements allow for print speeds exceeding 500 mm/s, while maintaining part accuracy and interlayer bonding strength, critical factors for composite filament applications. The integration of active vibration compensation, real-time extrusion monitoring, and automated bed leveling further improves print repeatability and reliability, addressing key concerns associated with rapid material deposition in high-speed printing environments [36].

One of the fundamental innovations enabling high-speed FFF printing is the development of high-flow extruders and all-metal hotends, designed to handle increased filament throughput without compromising extrusion consistency [109]. Contemporary FFF 3D printers, for instance, incorporate a hardened steel drive system to withstand abrasive composite filaments, such as carbon-fiber-reinforced PLA and PETG, ensuring minimal nozzle wear over extended print cycles. Additionally, direct drive extruders with precision gear ratios improve filament feeding and retraction response times, mitigating issues like stringing and under-extrusion that commonly arise at elevated print speeds. Other such FFF 3D printers utilize dual extrusion capabilities to facilitate multi-material printing, allowing for the integration of soluble support materials (e.g., PVA or BVOH), which is particularly beneficial for producing complex drone structures with internal cavities or embedded electronic channels [110,111,112,113].

Beyond hardware enhancements, advancements in firmware algorithms and slicing strategies have played a pivotal role in optimizing high-speed FFF performance. Modern slicing software, such as OrcaSlicer [114] and UltiMaker Cura (Ultimaker, Utrecht, The Netherlands) [115], now includes dynamic acceleration and jerk control, adapting motion parameters in real time to maintain print quality while maximizing speed. The implementation of thermal compensation models prevents warping and delamination by fine-tuning extrusion temperatures and cooling rates based on the specific thermal properties of composite-reinforced filaments. These innovations collectively enable the fabrication of lightweight, high-strength drone components with improved mechanical anisotropy and dimensional stability, paving the way for further integration of 3D printing in the rapid prototyping and production of UAV structures.

### 4.2. Influence of Increased Speeds on Material Flow, Layer Adhesion, and Print Quality

The transition to high-speed FFF 3D printing introduces significant challenges in maintaining consistent material flow dynamics, as the rapid extrusion of filament places increased demands on the melt chamber’s thermal stability and the extruder’s feed control. At print speeds exceeding 300–500 mm/s, the viscosity and flow rate of polymer composites become critical factors in determining deposition uniformity. Insufficient heat transfer can result in under-extrusion, where the material fails to fully liquefy before reaching the nozzle, leading to incomplete bonding between adjacent lines. Conversely, excessive thermal buildup can cause over-extrusion, producing unwanted swelling and surface irregularities. To counteract these effects, contemporary FFF 3D printers employ high-flow hotends with extended melt zones, allowing filaments—especially composite materials like carbon-fiber-reinforced PETG and nylon—to reach a stable molten state before deposition [36].

Layer adhesion, a fundamental determinant of part strength, is significantly influenced by print speed, as shorter layer cooling times at high speeds can disrupt the formation of strong interlayer bonds [116]. In traditional FFF printing, polymer chains have sufficient time to diffuse across layer interfaces, promoting mechanical integrity. However, in high-speed printing, reduced dwell times between layers can lead to incomplete polymer entanglement, increasing the risk of delamination and interlayer void formation. To address this, modern high-speed printers integrate actively heated build chambers, maintaining an elevated ambient temperature to slow down cooling rates, thereby enhancing layer fusion. Additionally, variable fan cooling strategies are employed to balance rapid solidification for overhang stability with prolonged heat retention for layer adhesion [117]. These factors are especially crucial for composite filament applications in drone manufacturing, where anisotropic mechanical properties and stress concentrations at interlayer boundaries can significantly impact flight performance and structural durability.

Print quality at high speeds is further affected by inertia-driven motion artifacts, such as ringing (ghosting), layer shifts, and inconsistent extrusion widths, which arise from rapid directional changes during deposition [118]. High-speed 3D printers mitigate these effects using closed-loop stepper motors with real-time position correction, input shaping algorithms, and rigid frame constructions that dampen unwanted oscillations. For composite filament-based drone components, maintaining dimensional accuracy is particularly important, as deviations in part geometry can affect aerodynamic efficiency, weight distribution, and component fitment. Innovations in slicing optimization, including adaptive acceleration control and segmented print paths, help preserve fine details without sacrificing throughput [119]. Furthermore, multi-axis calibration systems ensure that tolerances remain within acceptable limits for load-bearing UAV structures, demonstrating that high-speed 3D printing can achieve both efficiency and precision in functional aerospace applications.

### 4.3. Role of Heated Build Chambers and Advanced Extruder Designs in Maintaining Composite Filament Integrity

The integrity of composite filaments, particularly those reinforced with carbon fiber, glass fiber, or Kevlar, is heavily influenced by the thermal environment in which they are processed [120]. Unlike standard thermoplastics, composite filaments exhibit higher melt viscosities and reduced thermal conductivity, making them more susceptible to warping, delamination, and inconsistent bonding when exposed to fluctuating ambient temperatures [121,122]. Heated build chambers play a crucial role in mitigating these issues by maintaining a controlled thermal environment, which reduces internal stresses and enhances interlayer adhesion. By keeping the chamber at an elevated temperature, residual thermal gradients are minimized, preventing premature cooling that could cause shrinkage-induced deformation [123]. In high-speed 3D printing, the integration of actively heated chambers enables composite filaments, such as nylon–carbon fiber blends, to maintain dimensional stability throughout the print cycle [124]. This is particularly advantageous for drone components, where tight tolerances and mechanical reliability are critical to ensure aerodynamic efficiency and structural integrity.

Beyond chamber heating, advanced extruder designs are fundamental to ensuring consistent material flow and filament integrity during high-speed FFF printing. Traditional extruders often struggle with composite materials due to the abrasive nature of fiber reinforcements, which accelerate nozzle wear and degrade extrusion precision over time [125]. To counteract this, hardened steel or ruby-tipped nozzles are now standard in high-performance systems, extending nozzle lifespan and preserving flow rate consistency. Additionally, modern high-speed printers feature direct-drive extruders with precision dual-gear feeding mechanisms, ensuring that composite filaments are transported smoothly without excessive bending or grinding [126]. These extruders are designed to maintain uniform filament tension, preventing slip or grinding issues that can disrupt deposition accuracy. Such a high-torque drive system provides superior grip and pressure regulation, essential for avoiding under-extrusion and weak interlayer bonds in drone frame fabrication [127].

**Figure 4:**
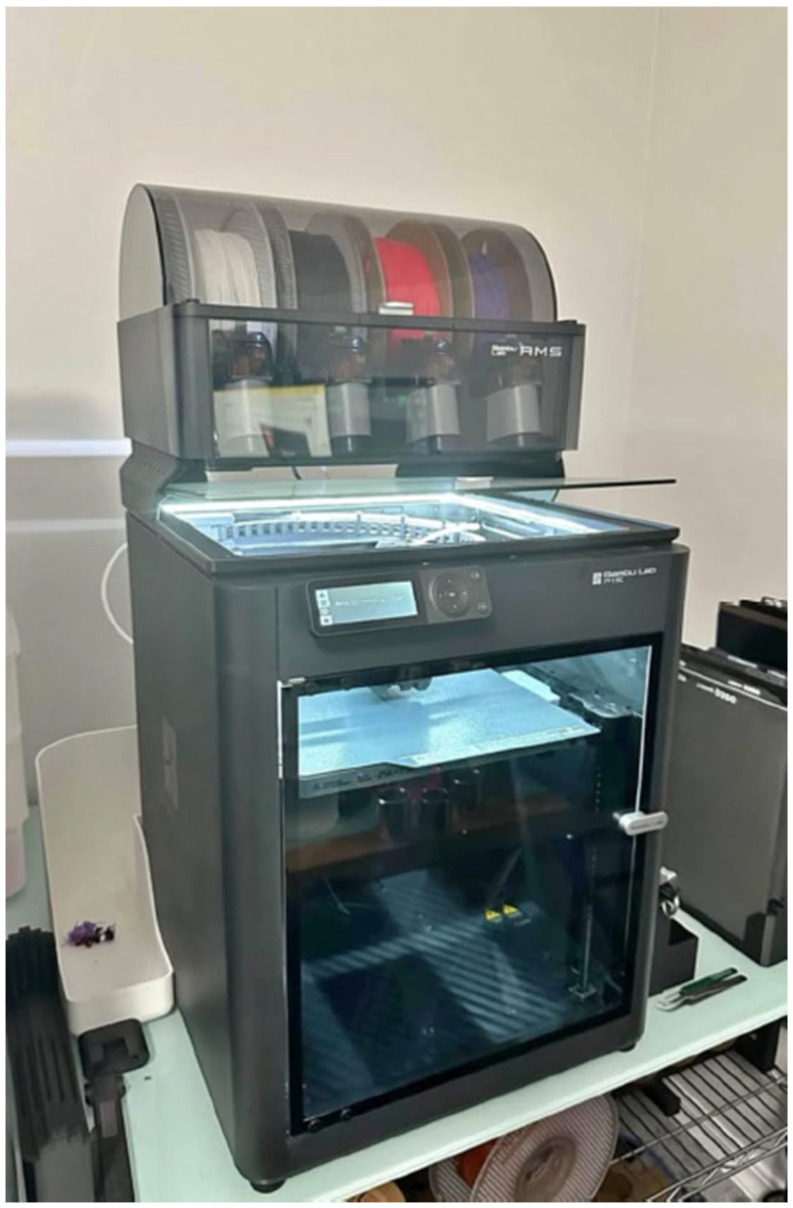
FDM 3D printer with enclosed chamber.

The thermal management of the extrusion process is another key aspect of maintaining composite filament integrity. Advanced extruders in high-speed FFF systems utilize all-metal hotends with extended melt zones, allowing composite filaments to reach their optimal extrusion temperature without premature solidification or degradation [128]. This is particularly beneficial for high-temperature engineering filaments like PEEK or PEI composites, which require precise thermal regulation to achieve proper fusion between deposited layers [129]. Furthermore, dynamic thermal monitoring sensors within these extruders actively adjust heating power based on real-time material flow data, preventing temperature fluctuations that could impact filament deposition [130,131]. The combination of heated build chambers, high-performance extruders, and intelligent thermal control systems ensures that composite filaments retain their structural integrity, mechanical strength, and dimensional accuracy, making high-speed 3D printing a viable solution for advanced UAV and aerospace applications [132]. Figure 4 depicts an FDM 3D printer with an enclosed chamber.

## 5. Embedded Electronics and Functional Integration

### 5.1. Soluble Support Materials for Complex Geometries

The use of soluble support materials in fused filament fabrication (FFF) 3D printing has become essential for producing complex, overhanging, and multi-material structures that would otherwise be impossible to fabricate with single-material printing. Soluble supports provide temporary structural reinforcement during printing but dissolve in water or mild solvents post-processing, eliminating the need for manual support removal, which can be time-consuming and risk damaging delicate features. Materials such as polyvinyl alcohol (PVA), butenediol vinyl alcohol copolymer (BVOH), and HydroFill are widely used due to their excellent solubility, adhesion properties, and thermal compatibility with engineering-grade thermoplastics [133]. These support materials are particularly beneficial for drone manufacturing, where aerodynamic surfaces, internal channels, and embedded electronics require intricate geometries that cannot accommodate conventional breakaway supports.

Among these materials, PVA remains the most commonly used soluble support due to its water solubility and good adhesion to standard printing filaments like PLA, PETG, and nylon [134]. However, it has limitations in humidity resistance and thermal stability, making it less effective for high-temperature composites. BVOH, a more advanced formulation, offers faster dissolution rates and improved moisture resistance, making it suitable for functional drone components requiring precision support removal in confined spaces [135]. Additionally, the relevant filament type with the commercial name “HydroFill”, designed specifically for engineering applications, exhibits enhanced thermal resistance and broader material compatibility, allowing for supporting complex geometries in high-strength composites like carbon-fiber-infused nylon [136]. These advancements enable multi-material 3D printing, where soluble supports allow for the seamless integration of structural, aerodynamic, and functional components in UAV frames.

The integration of soluble supports into high-speed FFF printing workflows has also required optimization of extrusion parameters, support interface strategies, and dissolution processes. Dual-extrusion 3D printers utilize independent material pathways to deposit precisely controlled support structures that adhere securely to printed parts without excessive material bleeding or interface defects [137]. Moreover, automated support generation algorithms in slicing software now incorporate adaptive support density and dissolution-optimized infill patterns, reducing both material consumption and post-processing time [138]. For UAV fabrication, this translates to lighter, more aerodynamic, and structurally efficient components, as internal cavities and aerodynamic features can be printed without the need for manual post-processing interventions, preserving design integrity and mechanical performance.

### 5.2. Techniques for Integrating Wiring Channels, Antennae, and Sensor Mounts Within Printed Drone Frames

Integrating wiring channels, antennae, and sensor mounts within 3D-printed drone frames requires an advanced understanding of additive manufacturing design principles, material properties, and functional optimization [139]. One of the key advantages of 3D printing in drone fabrication is the ability to create intricate internal structures that enhance performance while minimizing weight. Wiring channels are crucial for protecting and organizing electrical components within the drone frame. By embedding these channels directly into the print design, engineers can ensure a streamlined and aerodynamically efficient structure without compromising accessibility. Techniques such as internal conduits, split-frame designs, and modular wiring enclosures enable the routing of power and signal cables while mitigating electromagnetic interference (EMI) through isolated pathways [140]. The use of high-performance thermoplastics like nylon and carbon-fiber-infused composites provides both mechanical robustness and electrical insulation. Additionally, integrating strain relief features and flexible conduits within the wiring channels can enhance durability, particularly in high-vibration environments where wire fatigue is a concern. Advanced approaches, such as conductive traces printed directly into the frame using conductive filaments or coatings, further reduce the need for excessive cabling while enhancing structural efficiency.

The placement and integration of antennae in 3D-printed drone frames play a critical role in ensuring optimal signal reception, minimizing interference, and maintaining communication reliability [141]. Traditional mounting solutions often involve externally attached antennae, which can introduce aerodynamic drag and susceptibility to mechanical damage. In contrast, 3D printing allows for the creation of embedded antenna slots and RF-transparent housings that optimize transmission performance while preserving the structural integrity of the frame [142]. Materials such as PLA and ABS, which do not interfere with radio frequencies, are commonly used in areas surrounding the antenna to minimize signal attenuation. For drones equipped with multiple antennae—such as MIMO (Multiple-Input, Multiple-Output) systems—strategic spacing and polarization alignment are essential to avoid signal cancelation and optimize data transmission [143]. Additionally, routing coaxial cables through dedicated channels within the frame helps protect them from wear and reduces interference from nearby electronic components. In high-performance UAVs, the integration of printed antennae using conductive inks or filaments offers a promising avenue for reducing weight and improving form factors, making the antenna an integral part of the drone’s frame rather than an external add-on.

Sensor integration within 3D-printed drone frames is another crucial aspect of enhancing UAV functionality, particularly for applications involving aerial imaging, LiDAR mapping, environmental monitoring, and navigation [144]. Properly designed sensor mounts must ensure stability, reduce vibration-induced errors, and maintain clear lines of sight for optical and ranging instruments. Techniques such as embedded mounting slots, vibration-dampening TPU (thermoplastic polyurethane) enclosures, and modular sensor pods allow for secure attachment while facilitating easy replacement or upgrades. Thermal considerations are also essential, particularly for sensors sensitive to heat, such as infrared cameras or inertial measurement units (IMUs). By incorporating passive cooling features like ventilation channels or heat sinks within the printed frame, designers can mitigate temperature-related performance degradation. Furthermore, adaptive mounting solutions, such as adjustable sensor brackets or gimbal-compatible fixtures, enhance the versatility of drone configurations, allowing them to adapt to diverse operational environments. Emerging advancements in multi-material 3D printing further expand these possibilities, enabling the direct integration of electrically conductive pathways for sensor communication, lightweight shielding for electromagnetic compatibility, and even shape-memory polymers for reconfigurable sensor mounts [145].

The seamless integration of wiring channels, antennae, and sensor mounts within 3D-printed drone frames exemplifies the growing sophistication of additive manufacturing in UAV design [146]. By utilizing multi-material printing, optimized geometries, and advanced material properties, engineers can achieve a high level of functional integration while maintaining structural efficiency and minimizing weight. The ability to embed wiring channels within the drone’s frame not only improves aerodynamics but also enhances durability by reducing exposure to environmental stressors. Similarly, the strategic placement of antennae within RF-transparent housings maximizes signal strength while mitigating interference, ensuring reliable communication and control [147]. Sensor mounts, designed with precision and vibration-dampening features, further extend the drone’s capabilities in data acquisition and navigation. As 3D printing technologies progress, innovations such as conductive traces, embedded electronics, and adaptive mounting systems will further assist UAV manufacturing, leading to smarter, lighter, and more resilient drone architectures optimized for diverse operational demands. Table 3 provides a structured comparison of methods for embedding electronics in 3D-printed UAV frames, assisting readers to select the best approach based on design constraints and functionality.

## 6. Challenges and Limitations

### 6.1. Material Processing Difficulties: Nozzle Wear, Anisotropy, and Warping Issues

Material processing difficulties in the 3D printing of composite filaments, such as nozzle wear, anisotropy, and warping, are significant challenges that can affect both the quality of printed parts and the longevity of the printing equipment. One of the most common issues in printing with fiber-reinforced filaments, especially carbon fiber and glass fiber composites, is nozzle wear [148]. The presence of abrasive fibers within the filament can lead to accelerated degradation of the nozzle, particularly when using standard brass nozzles. This wear can result in inconsistent extrusion, reduced print quality, and even complete failure of the printing process if the nozzle becomes severely damaged. To mitigate nozzle wear, it is often recommended to use hardened steel or other wear-resistant materials for the nozzle, although these alternatives may still require regular maintenance and calibration to ensure smooth operation. Furthermore, the abrasive nature of fiber-reinforced filaments can contribute to other issues, such as filament jamming or clogging, particularly if the filament is not adequately mixed or the fibers are not uniformly distributed [149]. These challenges necessitate careful consideration of the choice of nozzles and the material quality to maintain both print performance and equipment longevity.

Anisotropy, the variation in material properties in different directions, is another critical issue that can arise when 3D printing with composite filaments [150]. Due to the layer-by-layer deposition process inherent in FDM/FFF 3D printing, the mechanical properties of the printed part can differ significantly between the horizontal and vertical directions, leading to anisotropic behavior. In parts printed with fiber-reinforced filaments, this anisotropy is often exacerbated by the alignment of the fibers during the printing process [151]. For example, fibers in a horizontal layer are typically aligned parallel to the print surface, leading to stronger mechanical properties in the same plane but weaker performance when subjected to forces perpendicular to the layers. This can be particularly problematic for parts that require uniform strength and stiffness in all directions, such as structural components in aerospace or automotive applications [152]. To address this issue, modifications in printing parameters, such as adjusting layer orientation, optimizing fiber alignment, or using multiple print directions, can help improve the material’s overall isotropy. Lastly, warping remains a prevalent issue when printing with composite filaments, especially in high-strength materials like carbon-fiber-reinforced PLA or nylon. As these materials cool, they can shrink unevenly, causing deformation and separation from the print bed. Warping is particularly problematic in larger parts or intricate geometries, where the differential cooling rates between the printed layers lead to stresses that cause the edges to lift. Using a heated print bed, controlling ambient temperature, or utilizing specialized adhesion techniques such as raft or brim printing are some strategies used to minimize warping and ensure dimensional accuracy in the final part [153].

### 6.2. Cost and Scalability of High-Performance Composite Filaments

The cost and scalability of high-performance composite filaments are critical factors that influence their adoption in both industrial and consumer-level 3D printing applications [154]. High-performance composite filaments, such as carbon fiber, glass fiber, and Kevlar-infused materials, are typically more expensive than standard thermoplastics due to the higher cost of raw materials and the complex manufacturing processes involved in their production. The incorporation of fibers into the base polymer not only increases material costs but also adds to the production complexity, as these filaments often require specialized equipment for extrusion and more stringent quality control to ensure uniform fiber dispersion [155]. Additionally, fiber-reinforced filaments are usually more abrasive than traditional filaments, necessitating the use of more durable, and therefore more expensive, print heads and nozzles. As a result, the upfront cost of using high-performance composite filaments can be a significant barrier, particularly for small-scale manufacturers, hobbyists, or startups with limited budgets. Moreover, the higher material costs often translate into increased costs per part printed, which can be prohibitive for low-margin industries or applications that require large quantities of parts [156].

Scalability, on the other hand, is a key consideration when integrating high-performance composite filaments into large-scale manufacturing processes [157]. While these materials offer enhanced mechanical properties, their cost-effectiveness diminishes as production volume increases unless the manufacturing process can be optimized for higher output. The relatively low throughput of high-performance filaments compared to standard thermoplastics can limit their use in mass production scenarios, where speed and cost per unit are essential factors [158]. Additionally, the need for specialized equipment, such as wear-resistant nozzles or high-temperature extruders, further complicates the scalability of these materials. However, ongoing advancements in 3D printing technology, including the development of high-speed printers and more efficient production techniques, have the potential to reduce the cost and improve the scalability of composite filament printing. For instance, innovations in multi-material printing, automation, and improved filament extrusion techniques could lead to greater efficiency and lower per-part costs [159]. In the long term, as demand for advanced materials grows and production scales up, economies of scale may help bring down the overall cost of high-performance composite filaments, making them more accessible for widespread industrial use.

While scalability is an essential advantage of additive manufacturing, the cost of composite filament materials and associated equipment remains a critical consideration. Carbon-fiber-infused PLA, PETG, or nylon filaments typically cost between 60 and 150% more than their unreinforced counterparts, primarily due to the incorporation of high-performance fillers and more complex production processes [160]. Additionally, high-speed 3D printers capable of processing these abrasive materials require hardened steel or ruby nozzles, advanced motion systems, and sometimes heated enclosures—all of which increase initial capital expenditure [161]. However, these costs are often offset by reduced lead times, minimized material waste, and the ability to produce functional drone components without the need for molds or post-processing. Furthermore, the potential for distributed, on-demand manufacturing offers long-term savings in logistics and inventory management. As the technology matures and economies of scale improve, the overall cost-efficiency of composite-based 3D printing is expected to become increasingly competitive with conventional production methods.

### 6.3. Reliability Concerns: Print Consistency, Delamination Risks, and Quality Control

Reliability concerns in 3D printing with high-performance composite filaments, including print consistency, delamination risks, and quality control, are significant challenges that can impact the overall performance and structural integrity of printed parts. Print consistency refers to the ability to produce parts with uniform quality and dimensions throughout the printing process [162]. Inconsistent extrusion, due to fluctuating filament diameters, variations in material viscosity, or improper handling of the filament, can lead to issues such as under-extrusion, over-extrusion, or inconsistent layer bonding. These inconsistencies can result in weak spots, dimensional inaccuracies, or surface imperfections, which can compromise the mechanical properties of the final part. Additionally, fiber-reinforced filaments are more prone to variations in the printing process due to their abrasive nature, which can lead to nozzle clogging or increased wear on printing components, further contributing to print variability [163]. Achieving consistent quality requires careful monitoring of print parameters such as extrusion temperature, print speed, layer height, and cooling rates, as well as the use of high-quality filament and well-maintained equipment.

Delamination risks are another critical reliability concern in 3D printing with composite filaments [164]. Delamination occurs when the layers of a printed part fail to properly bond, resulting in weak interfaces that can lead to part failure under stress. This issue is particularly prevalent in composite filaments due to fiber reinforcement, which can create anisotropic bonding behavior between the polymer matrix and the reinforcing fibers [165]. Factors such as insufficient extrusion temperature, improper layer adhesion, or rapid cooling can exacerbate delamination. In high-performance filaments like carbon fiber and glass fiber composites, the layers may also experience different rates of thermal expansion, further increasing the risk of separation between layers. Delamination can significantly undermine the strength and durability of the printed part, particularly in load-bearing applications. Ensuring proper layer adhesion requires precise control over print parameters, including maintaining an optimal printing temperature and cooling rate, as well as selecting suitable printing substrates that promote adhesion [166].

In addition to delamination and layer adhesion inconsistencies, uneven cooling and thermal contraction remain critical issues affecting the dimensional accuracy and mechanical reliability of 3D-printed composite parts. As layers cool at different rates, internal stresses can develop, resulting in warping, shrinkage, or distortion of the printed geometry [167]. These thermal gradients can also reduce adhesion to the build platform, leading to part detachment or surface defects during the early stages of printing. Moreover, the interaction between reinforced fibers and the polymer matrix during solidification can amplify these effects due to differing thermal expansion coefficients. Another source of reliability concern lies in the variations in filament quality and extrusion parameters—such as temperature, flow rate, and print speed—which can lead to inconsistent layer deposition, void formation, and surface roughness. Rapid cooling, especially in high-speed printing scenarios, may hinder proper polymer chain diffusion across layers, producing weak interlayer bonds prone to failure under dynamic or cyclic loading [168]. These failure mechanisms are particularly detrimental in drone applications, where lightweight structures must endure significant mechanical stress and vibration. To mitigate such risks, careful thermal management, controlled cooling environments, and rigorous filament quality assurance are essential, alongside real-time monitoring of extrusion conditions to maintain print consistency and mechanical integrity.

Quality control is essential to ensure that composite filaments produce reliable, high-performance parts. Without rigorous quality control measures, inconsistencies in material composition, fiber dispersion, and bonding can lead to variations in mechanical properties, compromising part performance [169]. Even slight variations in the filament, such as uneven fiber distribution or poor polymer matrix formulation, can result in parts with inferior mechanical properties or unexpected behavior under load. To address these concerns, manufacturers must implement strict quality control processes, such as material inspection, batch testing, and post-processing evaluations, to verify that the composite filaments meet the required standards for strength, durability, and consistency. Advanced testing methods, such as tensile testing, impact resistance analysis, and microscopic examination of fiber dispersion, can help identify potential weaknesses early in the manufacturing process [170]. Furthermore, the use of automated monitoring systems and real-time quality assessments during the printing process can help mitigate these reliability concerns, ensuring that each part meets the necessary specifications for its intended application [170].

In conclusion, the challenges associated with material processing, cost, scalability, and reliability in the use of high-performance composite filaments highlight the complexities of integrating these advanced materials into 3D printing applications. While these filaments offer significant advantages in terms of strength, weight optimization, and functionality, their successful adoption requires overcoming hurdles such as nozzle wear, delamination risks, and maintaining consistent print quality. Furthermore, the cost of high-performance materials and the scalability of the production process remain critical considerations for their widespread use, particularly in industrial settings. As 3D printing technologies progress, solving these obstacles through improved materials, optimized processing techniques, and enhanced quality control will be paramount to utilizing composite filaments for a broad range of applications, including drone manufacturing. Table 4 provides a comprehensive overview of the main challenges encountered when using composite filaments in drone manufacturing, while also outlining practical mitigation strategies that can help improve print quality and reliability.

## 7. Future Prospects and Research Directions

### 7.1. Innovations in Nanocomposite Filaments

As 3D printing technology progresses, the development of next-generation composite filaments will greatly aid drone manufacturing by enhancing mechanical properties, weight efficiency, and functional integration [170,171]. Traditional carbon-fiber-infused polymers have already demonstrated significant improvements in strength-to-weight ratios, but emerging nanocomposite materials—such as graphene and carbon nanotube (CNT)-reinforced polymers—offer even greater potential for lightweight, high-strength, and multifunctional UAV components [172]. These nanomaterials introduce superior electrical conductivity, thermal stability, and impact resistance, opening new possibilities for self-sensing, self-healing, and energy-efficient drone structures. Moreover, advancements in artificial intelligence-driven topology optimization and hybrid manufacturing techniques—which combine additive manufacturing with CNC machining—are expected to push the boundaries of structural performance and design flexibility. This chapter explores key innovations shaping the future of 3D-printed drone components, focusing on nanocomposite filaments, AI-assisted design strategies, and hybrid fabrication approaches that will drive the next generation of high-performance UAVs.

The integration of nanocomposites into 3D-printed drone components represents a major leap forward in material performance, particularly in terms of mechanical strength, electrical conductivity, and thermal stability [173]. Among the most promising advancements are graphene-enhanced filaments, which exhibit exceptional stiffness-to-weight ratios, high electrical conductivity, and superior thermal dissipation properties. The incorporation of graphene nanoplatelets (GNPs) into thermoplastic matrices such as PLA, PETG, and nylon has demonstrated notable improvements in fracture toughness and tensile modulus, making them ideal for lightweight yet robust UAV structures [174]. Furthermore, the intrinsic electrical conductivity of graphene-infused filaments allows for the potential fabrication of self-sensing and self-heating drone components, reducing the need for additional wiring or embedded electronic systems [175]. Despite these advantages, dispersion challenges and interfacial bonding limitations between graphene and polymer matrices remain key hurdles that require further research. Advances in functionalized graphene derivatives and novel extrusion techniques, such as shear-mixing twin-screw compounding, could mitigate these issues, leading to more homogeneous and high-performance graphene-based filaments suitable for drone applications.

Similarly, carbon nanotube (CNT)-reinforced polymers are emerging as a game-changing class of nanocomposite filaments for additive manufacturing of UAV components. CNTs possess ultrahigh aspect ratios, remarkable tensile strength, and excellent electrical and thermal conductivity, enabling the development of high-performance drone frames and functional parts [176,177]. The incorporation of CNT networks within high-temperature thermoplastics such as PEEK and PEI can lead to unprecedented improvements in impact resistance, damping properties, and fatigue life, making these materials particularly attractive for drones operating in extreme conditions. Additionally, the electrical conductivity of CNT-reinforced filaments opens new possibilities for directly printed EMI shielding components, lightweight antenna structures, and integrated sensor arrays within UAV frames [178]. However, similar to graphene, CNT dispersion and alignment within polymer matrices present significant processing challenges [179]. Future research must focus on optimized printing parameters, surface-functionalized CNTs, and multi-scale hybrid reinforcements (e.g., CNT/graphene hybrids) to maximize structural integrity and multifunctional properties in 3D-printed drone applications.

### 7.2. AI-Driven Topology Optimization for Lightweight Drone Frames

The integration of artificial intelligence (AI) and machine learning (ML) in topology optimization is transforming the design and fabrication of lightweight drone frames, enabling structures that maximize mechanical performance while minimizing material usage [180]. Traditional design approaches often rely on intuitive engineering principles or finite element analysis (FEA) to enhance aerodynamic efficiency and structural integrity. However, AI-driven topology optimization leverages evolutionary algorithms, generative design, and neural networks to explore a vast design space beyond human intuition, identifying highly optimized load-bearing structures with intricate biomimetic geometries. By training ML models on extensive datasets of stress distributions, vibrational modes, and failure patterns, AI can generate lightweight, high-stiffness geometries that are ideally suited for additive manufacturing [181]. This computational approach is particularly valuable for 3D-printed UAV frames, where anisotropic properties, layer adhesion constraints, and print path orientation must be carefully considered to ensure optimal strength-to-weight ratios. Recent advancements in physics-informed AI models further refine this process, enabling the prediction of multi-material interactions and the adaptive reinforcement of critical load zones, ultimately leading to more efficient and structurally robust drone architectures [182].

Apart from static weight reduction, AI-driven topology optimization is also facilitating multi-functional drone designs that integrate embedded electronics, energy storage elements, and aerodynamic enhancements [183]. For instance, bioinspired lattice structures and functionally graded materials (FGMs)—generated through AI algorithms—can be strategically placed within a drone’s frame to optimize energy absorption and impact resistance, reducing failure risks in crash scenarios. Moreover, AI-driven real-time optimization loops allow for on-the-fly adjustments to UAV structures based on operational feedback, leading to the fabrication of self-adapting, reconfigurable drones [184]. This is particularly advantageous for high-speed FFF 3D printing, where printability constraints and material flow dynamics can be incorporated into iterative AI-driven simulations to refine the design-for-manufacturability process [185]. As AI models become more advanced, the future of topology optimization for drone fabrication will likely involve integrated AI-CAD environments, where design, simulation, and production constraints are holistically managed, leading to autonomous, AI-generated UAV structures with elevated efficiency, durability, and mission adaptability.

### 7.3. Hybrid Manufacturing Approaches: 3D Printing and CNC Machining for Performance Enhancement

Also, a potential combination of additive manufacturing (AM) and subtractive techniques such as CNC machining could prove very useful in the fabrication of high-performance drone components [186]. While 3D printing enables the rapid prototyping of complex geometries with minimal material waste, it often faces limitations in surface finish, dimensional accuracy, and material properties, particularly in the context of high-performance UAV applications. By integrating CNC machining into the post-processing workflow, hybrid manufacturing approaches can significantly enhance the precision, mechanical integrity, and functional capabilities of 3D-printed drone structures [187]. For instance, fused filament fabrication (FFF) and vat photopolymerization (VPP) processes can be used to produce near-net-shape UAV components, which are subsequently refined through CNC milling to achieve tighter tolerances, smoother surfaces, and improved aerodynamic efficiency. This is particularly relevant for load-bearing elements such as drone arms, motor mounts, and structural frames, where surface defects or layer anisotropy from additive processes can introduce stress concentrations that compromise durability. Additionally, hybrid manufacturing enables the strategic reinforcement of critical regions by machining precise pockets or cavities for embedding high-strength inserts, such as carbon fiber or titanium reinforcements, thus optimizing weight-to-strength ratios without sacrificing performance [188]. This synergy between AM and CNC machining addresses the inherent trade-offs in both processes, enabling UAVs to achieve superior structural integrity, flight stability, and functional versatility.

Beyond improving dimensional accuracy and mechanical properties, hybrid manufacturing also enhances the material and design freedom available for UAV fabrication. While traditional CNC machining is often constrained by tool accessibility and material removal limitations, 3D printing allows for the creation of intricate internal features, such as lattice structures, cooling channels, and sensor integration cavities, that would be challenging or impossible to machine conventionally [189]. By utilizing a hybrid approach, engineers can optimize UAV components for specific performance criteria, such as enhanced thermal dissipation for electronic housings, lightweight energy-absorbing frames for impact resistance, or aerodynamic wing structures with high lift-to-drag ratios. Additionally, hybrid techniques facilitate multi-material integration, where 3D-printed polymer or composite structures can be seamlessly combined with machined metal components, enabling the production of hybrid UAV airframes that balance weight, strength, and functional adaptability [190]. Emerging advancements in automated toolpath generation and AI-driven process planning are further streamlining the integration of AM and CNC machining, allowing for adaptive manufacturing workflows where material deposition and precision machining are dynamically adjusted based on in situ sensor feedback [191]. This combination of additive and subtractive processes is undoubtedly leading to the introduction of next-generation UAVs with unprecedented levels of efficiency, durability, and mission-specific customization, making hybrid manufacturing a promising perspective of future drone production.

## 8. Conclusions

The integration of composite filaments in 3D printing technologies has significantly enhanced the fabrication of UAV components by improving strength-to-weight ratios, structural integrity, and aerodynamic efficiency. The study underscores the advantages of carbon-fiber-infused PLA, PETG, and nylon in producing lightweight, high-performance drone parts, with high-speed FFF printing and soluble support materials further expanding design possibilities. Despite the benefits, challenges such as nozzle wear, anisotropic mechanical properties, and scalability issues remain obstacles to widespread adoption. Solutions such as optimized fiber dispersion, advanced extruder designs, and hybrid manufacturing techniques combining additive and subtractive methods are critical in addressing these limitations. Additionally, the integration of embedded electronics and sensor mounts through additive manufacturing opens new opportunities for functional UAV applications, including real-time data acquisition and autonomous operations. Looking ahead, innovations in nanocomposite materials, AI-assisted structural optimization, and multi-material 3D printing will play a crucial role in advancing drone manufacturing. By overcoming existing limitations and utilizing cutting-edge technologies, 3D printing is believed to become an enabling technology for next-generation UAV development, leading to the fabrication of more efficient, customizable, and high-performance aerial systems.

## Figures and Tables

**Table 1:** Classification of composite filaments used in 3D printing: key properties and general applications across industries.

Reinforcing Material	Base Polymer	Key Properties	General Applications
Carbon Fiber	PLA, PETG, Nylon	High strength-to-weight ratio, excellent rigidity, thermal stability	Aerospace, automotive components, structural parts, sports equipment
Glass Fiber	PLA, PETG, ABS	High impact resistance, increased stiffness, good durability	Automotive parts, construction materials, industrial applications, protective housings
Kevlar	PLA, Nylon, PETG	High abrasion resistance, flexibility, toughness, vibration damping	Protective gear, bulletproof vests, automotive parts, industrial belts
Hybrid (e.g., Carbon Fiber and Glass Fiber)	PLA, PETG, Nylon	Balanced strength, impact resistance, and thermal stability	Robotics, aerospace, automotive, structural components requiring multi-property optimization
Aramid Fiber	PLA, PETG	Lightweight, high strength, corrosion resistance, toughness	Military applications, protective wear, marine, and aerospace industries

**Table 2:** Comparative mechanical properties of composite filaments for 3D-printed drone parts. Data are compiled from the aforementioned published literature and manufacturer specifications to provide an overview of typical performance ranges for each material type.

Filament Type	Tensile Strength (MPa)	Young’s Modulus (GPa)	Impact Resistance (kJ/m^2^)	Density (g/cm^3^)	Key Applications in Drones
Carbon Fiber-PLA	60–80	4.5–6.0	4–6	1.3–1.4	Drone frames, motor mounts, structural parts
Carbon Fiber-PETG	50–70	3.0–4.5	8–12	1.3–1.4	Propellers, landing gear, aerodynamic components
Carbon Fiber-Nylon	80–110	6.5–8.0	10–15	1.1–1.2	High-stress drone arms, propeller guards
Glass Fiber-PETG	40–60	3.0–5.0	12–16	1.4–1.5	Protective covers, structural reinforcement
Kevlar-PLA	45–65	3.5–5.0	15–18	1.2–1.3	Impact-resistant housings, protective enclosures
Hybrid (Carbon and Glass Fiber-PLA)	70–90	5.5–7.0	8–10	1.3–1.5	Structural frames requiring high stiffness and toughness

**Table 3:** Summary of structural performance characteristics of 3D-printed drone components made from composite filaments. Values are synthesized from the aforementioned published literature sources to highlight key trends and applications.

Integration Method	Description	Advantages	Challenges	Applications in Drones
Embedded Wiring Channels	Pre-designed internal pathways for routing cables.	Protects wires from damage, reduces drag, improves aerodynamics.	Requires precise design and printing; limited flexibility for modifications.	Power distribution, motor wiring, ESC connections.
Printed Conductive Traces	Conductive inks or filaments used to print circuits directly into the frame.	Eliminates excess wiring, lightweight, allows for custom circuitry.	Limited conductivity, requires special materials and post-processing.	Signal routing, integrated power distribution.
Soluble Support-Based Conduits	Temporary support material is used to create hollow channels for wiring.	Enables complex internal structures, improves esthetics.	Requires dual extrusion printers, post-processing needed.	Sensor integration, embedded cabling.
RF-Transparent Antenna Housings	Specialized non-conductive enclosures to improve signal strength.	Reduces interference, protects delicate antenna structures.	Material selection critical for performance; positioning must be optimized.	GPS, telemetry, communication antennas.
Integrated Sensor Mounts	Custom-designed slots or enclosures for UAV sensors.	Secure sensor placement, minimizes vibrations.	Requires careful calibration to prevent misalignment.	Camera mounts, LiDAR, IMU, thermal imaging sensors.
Multi-Material Printing for Embedded Electronics	Uses conductive and insulating materials to embed electronics.	Allows functional parts to be directly printed with electronics.	High material costs, requires advanced multi-material printers.	Smart drone frames, real-time health m

**Table 4:** Challenges, impact, and mitigation strategies in composite filament 3D printing for drones.

Challenge	Description	Impact on Drone Components	Mitigation Strategies	Comments
Nozzle Wear	Abrasive fibers (e.g., carbon/glass) accelerate nozzle degradation.	Inconsistent extrusion, reduced print quality, increased downtime.	Use wear-resistant nozzles (hardened steel, ruby-tipped); regular maintenance.	Essential for maintaining consistent material flow.
Anisotropy	Layer-by-layer deposition causes directional dependency in mechanical properties.	Weakened interlayer bonds may lead to delamination under stress.	Optimize print orientation, adjust fiber alignment, use multi-directional printing strategies.	Critical for load-bearing parts where uniform strength is required.
Warping	Uneven cooling and thermal contraction lead to deformation.	Dimensional inaccuracies, poor adhesion to build platform, structural weaknesses.	Use heated build plates, controlled ambient temperature, rafts/brims for better adhesion.	Especially problematic for larger or intricate components.
Print Consistency	Variations in filament quality, extrusion temperature, or speed.	Inconsistent mechanical properties, surface imperfections, potential failure points.	Implement rigorous quality control, real-time monitoring systems, and standardize filament production.	Consistency is key to ensuring reliable drone performance.
**Delamination Risks**	Inadequate bonding between layers due to rapid cooling or poor adhesion.	Structural failure under dynamic loading conditions.	Fine-tune printing parameters (temperature, speed, cooling rates); use optimized bonding techniques.	Requires careful calibration, especially for high-stress parts.
**Cost and Scalability**	High-performance composite filaments and specialized equipment are expensive.	Increased production cost, limited accessibility for small-scale manufacturers.	Economies of scale, process optimization, hybrid manufacturing (3D printing and CNC machining).	Balancing performance with cost remains a significant challenge.

## Data Availability

No new data were created or analyzed in this study. Data sharing is not applicable to this article.

## Abbreviations

The following abbreviations are used in this manuscript:

3D	Three-Dimensional
ABS	Acrylonitrile Butadiene Styrene
AI	Artificial Intelligence
AM	Additive Manufacturing
BVOH	Butenediol Vinyl Alcohol Copolymer
CAD	Computer-Aided Design
CFF	Continuous Fiber Fabrication
CFD	Computational Fluid Dynamics
CNT	Carbon Nanotube
CNC	Computer Numerical Control
EMI	Electromagnetic Interference
FFF	Fused Filament Fabrication
FGM	Functionally Graded Materials
FEA	Finite Element Analysis
FDM	Fused Deposition Modeling
GNP	Graphene Nanoplatelets
IMU	Inertial Measurement Unit
ML	Machine Learning
MIMO	Multiple-Input Multiple-Output
PA	Polyamide (Nylon)
PCA	Principal Component Analysis
PCL	Polycaprolactone
PCTG	Polycyclohexylenedimethylene Terephthalate Glycol
PEEK	Polyether Ether Ketone
PEI	Polyetherimide
PETG	Polyethylene Terephthalate Glycol
PLA	Polylactic Acid
PVA	Polyvinyl Alcohol
RF	Radio Frequency
SLA	Stereolithography
SLS	Selective Laser Sintering
TPU	Thermoplastic Polyurethane
UAV	Unmanned Aerial Vehicle
VPP	Vat Photopolymerization
